# Iatrogenic Pulmonary Artery Pseudoaneurysm Following Swan-Ganz Catheterization: Successful Endovascular Coil Embolization

**DOI:** 10.7759/cureus.111900

**Published:** 2026-07-01

**Authors:** Renato Abu Hana, Sebastian Jimenez Melendez, Kirolous Soliman, Grit A Adler, Vinicius Fornazari

**Affiliations:** 1 Interventional Radiology, University of Florida College of Medicine – Jacksonville, Jacksonville, USA; 2 Radiology, University of Florida College of Medicine – Jacksonville, Jacksonville, USA

**Keywords:** coil embolization, hemoptysis, interventional radiology, papa, pulmonary artery catheterization, pulmonary artery pseudoaneurysm, pulmonary hypertension, swan-ganz catheter

## Abstract

Pulmonary artery pseudoaneurysm (PAPA) is a rare but life-threatening complication of Swan-Ganz catheterization. We report the case of a 71-year-old woman with severe pulmonary hypertension, mechanical mitral valve replacement requiring chronic anticoagulation, and chronic obstructive pulmonary disease who developed moderate hemoptysis within hours of Swan-Ganz catheterization. Computed tomography angiography (CTA) demonstrated a 12 mm pseudoaneurysm arising from a subsegmental branch of the right middle lobe pulmonary artery with surrounding alveolar hemorrhage, absent on admission imaging. Interventional radiology performed successful endovascular coil embolization using a sandwich technique, with complete resolution of hemoptysis within 24 hours of the procedure. This case highlights the importance of recognizing PAPA as a cause of post-catheterization hemoptysis, the critical role of CTA in diagnosis, and the effectiveness of endovascular coil embolization as the preferred minimally invasive treatment approach.

## Introduction

The Swan-Ganz catheter, formally known as the pulmonary artery catheter (PAC), remains a valuable tool for hemodynamic monitoring in selected critically ill patients, providing direct measurement of pulmonary vascular pressures, cardiac output, and pulmonary capillary wedge pressure [1]. Despite a decline in its routine use, PAC placement remains indicated in the evaluation and management of pulmonary hypertension, cardiogenic shock, and complex cardiopulmonary disease [1,2]. In contemporary practice, the catheter is inserted via central venous access, most commonly through the internal jugular or subclavian vein, and advanced through the superior vena cava, right atrium, and right ventricle, into the pulmonary artery and its branches. At the bedside, venous access is obtained under ultrasound guidance, and catheter positioning is confirmed by sequential intravascular pressure waveform changes as the balloon-tipped catheter is floated forward through each cardiac chamber [1]. 

Swan-Ganz catheterization carries inherent procedural risk, with serious complications reported in up to 17% of insertions, including arrhythmias, thromboembolism, catheter knotting, infection, and vascular injury [3,4]. Among the most feared is pulmonary artery rupture with hemorrhage or pseudoaneurysm formation, with a reported incidence of 0.03-0.2% of all catheterizations and an associated acute mortality rate approaching 70% [4,5]. Pulmonary artery pseudoaneurysm (PAPA) represents a distinct but related entity: a contained vascular disruption in which mechanical injury to the vessel wall, from catheter tip migration into a distal branch, balloon overinflation, or eccentric inflation within a non-compliant vessel, creates a false sac bounded by perivascular tissue and thrombus rather than true vascular layers [6]. During routine catheter advancement, the balloon is inflated to wedge the tip into a distal pulmonary arterial branch; if the catheter has migrated too far distally or the vessel is rigid due to pulmonary hypertension, the outward radial force of balloon inflation can shear through the vessel wall, producing transmural disruption. The resulting false sac lies in direct proximity to pulmonary alveoli, such that its erosion or rupture permits blood to enter the airways, manifesting clinically as hemoptysis. This distinguishes PAPA from a true aneurysm, which involves dilation of all three vessel wall layers, and carries important implications for rupture risk and management. 

Several patient-level risk factors for catheter-induced pulmonary artery injury have been identified, including severe pulmonary hypertension, anticoagulation therapy, advanced age, and female sex [4,5,7]. Pulmonary hypertension is of particular relevance, as chronic exposure to elevated intravascular pressure induces vascular remodeling that renders arterial walls less compliant and more susceptible to mechanical disruption. Anticoagulation compounds this risk by impairing the physiologic tamponade effect of perivascular hematoma that would otherwise limit hemorrhage from a vascular tear [7]. 

Endovascular embolization has emerged as the preferred treatment for PAPA based on available evidence, which consists predominantly of case series and retrospective studies demonstrating high technical success rates and low procedural morbidity [8,9]. We report a case of iatrogenic PAPA following bedside Swan-Ganz catheterization, successfully treated with endovascular coil embolization by interventional radiology. 

## Case presentation

A 71-year-old woman with a medical history significant for severe pulmonary hypertension, mechanical mitral valve replacement on chronic warfarin anticoagulation, atrial fibrillation, chronic kidney disease stage 3b, chronic obstructive pulmonary disease on supplemental home oxygen, and bed-bound functional status presented with acute cardiopulmonary decompensation, maintaining her baseline oxygen requirements with stable vital signs on admission. 

Admission CT angiography (CTA) of the chest performed as a standard pulmonary embolism protocol with intravenous contrast, achieving adequate pulmonary arterial opacification, demonstrated no pulmonary embolism and no PAPA. Incidental mild dilation of the main pulmonary artery measuring 3.4 cm was noted, above the normal threshold of 2.9 cm [10], consistent with the known diagnosis of pulmonary hypertension. Transthoracic echocardiography revealed a preserved left ventricular ejection fraction of 60-65% with severe pulmonary hypertension, with an estimated systolic pulmonary artery pressure of approximately 72 mmHg. As echocardiography estimates pulmonary pressures indirectly and is subject to measurement variability, right heart catheterization was performed to confirm the diagnosis, characterize pre- versus post-capillary physiology, and guide therapeutic decision-making in accordance with current pulmonary hypertension guidelines [11]. 

The procedure was performed at the bedside using ultrasound guidance for right internal jugular venous access. A balloon-tipped, flow-directed Swan-Ganz PAC (Edwards Lifesciences, Irvine, CA, USA) was advanced using continuous intravascular pressure waveform monitoring alone, without fluoroscopic or chest radiographic guidance: catheter advancement was confirmed by sequential identification of right atrial, right ventricular, pulmonary artery, and pulmonary capillary wedge pressure waveforms, with wedge pressure confirmed by characteristic waveform dampening upon balloon inflation [12]. No technical difficulty was documented. Right heart catheterization confirmed severe pulmonary hypertension, with a mean pulmonary artery pressure of 39 mmHg (normal < 20 mmHg) and pulmonary capillary wedge pressure of 22 mmHg (normal 6-12 mmHg), consistent with combined pre- and post-capillary pulmonary hypertension. 

Within hours of catheterization, the patient developed new-onset moderate hemoptysis (estimated 30-100 mL over 24 hours), accompanied by worsening right-sided chest pain. Vital signs remained stable throughout, with no tachycardia, hypotension, oxygen desaturation, or significant decline in hemoglobin. The international normalised ratio (INR) at the time of hemoptysis was 2.8, within the therapeutic range for her mechanical mitral valve (target 2.5-3.5); anticoagulation was not reversed. Repeat CTA of the chest, performed two days after Swan-Ganz catheterization, demonstrated interval development of a new 12 mm pseudoaneurysm arising from a subsegmental branch of the right middle lobe pulmonary artery, with adjacent ground-glass opacity consistent with pulmonary hemorrhage, without pleural effusion or hemothorax (Figure 1), absent on imaging obtained at admission. Given the clear temporal relationship to Swan-Ganz catheterization, a diagnosis of iatrogenic PAPA was established, with severe pulmonary hypertension and therapeutic anticoagulation identified as contributing risk factors. 

**Figure 1:**
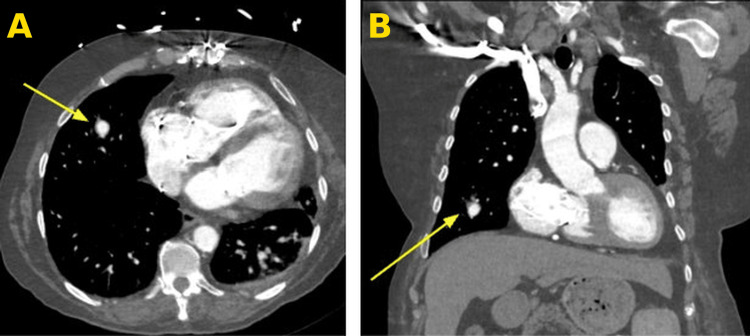
Axial (A) and coronal (B) contrast-enhanced CT angiography of the chest demonstrating a 12 mm pseudoaneurysm (arrow) arising from a subsegmental branch of the right middle lobe pulmonary artery, with adjacent ground-glass opacity consistent with alveolar hemorrhage. No pleural effusion or hemothorax is identified.

Interventional radiology was consulted, and the patient was brought to the angiography suite for endovascular treatment. Right common femoral venous access was obtained under real-time ultrasound guidance using a micropuncture kit, upsized to a 5-French vascular sheath. Using a Glidewire and angled pigtail catheter, nonselective pulmonary angiography was performed for vascular mapping. A 5-French Berenstein catheter (AngioDynamics, Inc., Latham, New York, United States) was then used to select the right interlobar pulmonary artery, and selective pulmonary angiography was performed. A 2.4-French Progreat microcatheter (Terumo Corporation, Tokyo, Japan) was advanced into sequential segmental branches of the right middle lobe pulmonary artery until angiographic opacification of the pseudoaneurysm sac was confirmed (Figure 2).

**Figure 2:**
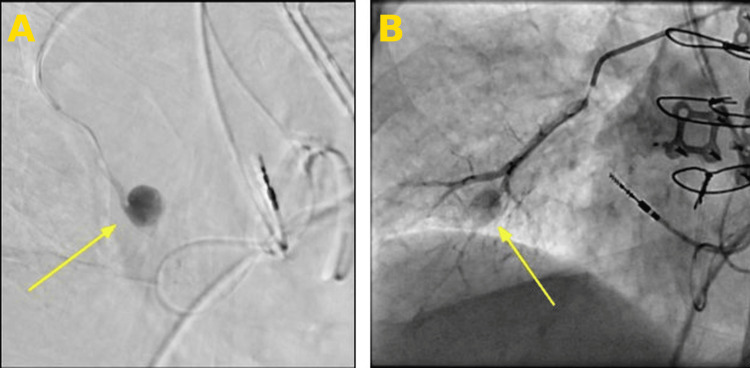
Selective pulmonary angiography of the right middle lobe pulmonary artery. (A) Digital subtraction angiography (DSA) demonstrating a well-defined, rounded pseudoaneurysm sac (arrow) arising from a subsegmental branch prior to embolization. (B) Microcatheter angiography confirming opacification of the pseudoaneurysm sac (arrow); selective injection into the target branch reproduced hemoptysis as an unintended observed finding, confirming catheterization of the feeding vessel.

Notably, selective injection into the target branch reproduced hemoptysis as an observed, unintended finding, likely secondary to pressurization of the pseudoaneurysm sac, at which point the injection was immediately discontinued, further confirming selective catheterization of the feeding vessel. Using a sandwich technique, the microcatheter was advanced into the pseudoaneurysm sac, and coil embolization was performed using five 4 mm × 80 mm Embold detachable coils (Boston Scientific Corporation, Marlborough, Massachusetts, United States), followed by embolization of the supplying segmental artery proximal and distal to the pseudoaneurysm sac. Parent artery sacrifice was considered acceptable given the subsegmental location of the feeding vessel, where embolization carries minimal risk of significant parenchymal ischemia. Post-embolization angiography demonstrated complete exclusion of the pseudoaneurysm with no residual opacification, preserved perfusion of adjacent pulmonary arterial branches, and no evidence of non-target embolization (Figure 3). Total fluoroscopy time was 26.9 minutes, with a total iodinated contrast volume of 180 mL. All catheters and sheaths were removed, and hemostasis was achieved with manual compression. 

**Figure 3:**
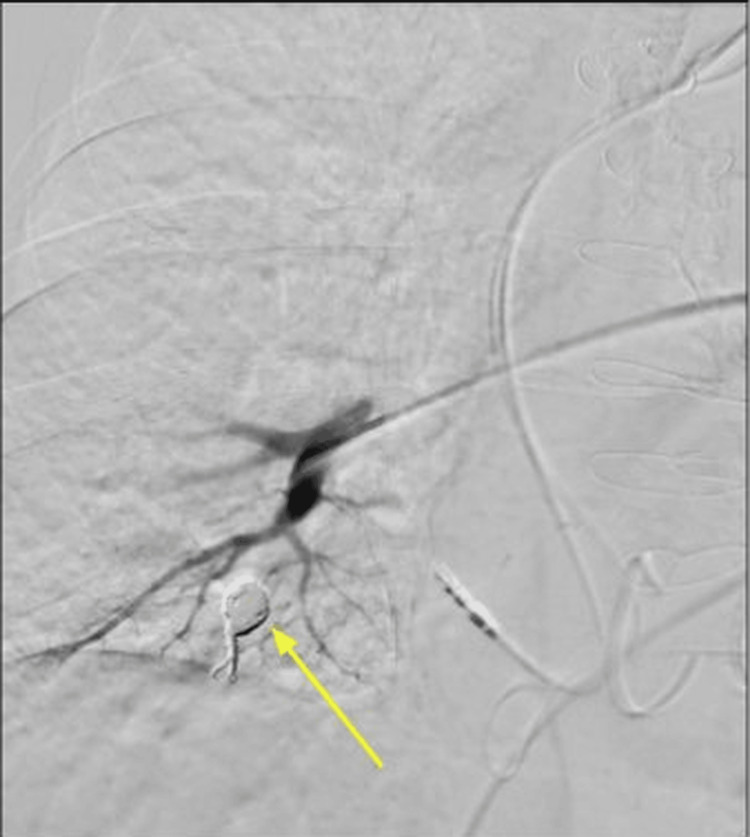
Post-embolization digital subtraction angiography (DSA) demonstrating successful coil embolization (arrow) with complete exclusion of the pseudoaneurysm, preserved perfusion of adjacent pulmonary arterial branches, and no non-target embolization.

Hemoptysis resolved within 24 hours of embolization, with no recurrence observed during the post-procedure observation period prior to discharge. Heparin infusion was resumed the following day, and the patient was transitioned back to warfarin with a target INR of 2.5-3.5 prior to discharge, without additional bridging. The patient was discharged in stable condition. Telephone follow-up conducted five days post discharge confirmed complete resolution of hemoptysis and chest pain. Repeat CT imaging was intentionally deferred given complete clinical resolution and to minimize radiation exposure and contrast nephropathy risk in the setting of chronic kidney disease stage 3b.

## Discussion

This case illustrates the diagnostic and therapeutic challenges of iatrogenic PAPA in a patient with multiple established risk factors for catheter-induced pulmonary artery injury. The presence of severe pulmonary hypertension, chronic anticoagulation, advanced age, and female sex collectively reflect a high-risk profile as described in the literature [4,5,7]. The rapid clinical deterioration following catheterization underscores the importance of a low threshold for repeat vascular imaging in this population. The initial admission CTA was negative - it was the interval development of hemoptysis in clear temporal relationship to catheterization that prompted repeat imaging, establishing the diagnosis. 

Catheter-induced PAPA typically arises through one of two mechanisms: distal migration of the catheter tip into a small-caliber peripheral pulmonary arterial branch, or eccentric balloon inflation within a vessel unable to accommodate the inflated diameter. Both generate shear forces against the vessel wall sufficient to produce transmural disruption, creating a contained false sac rather than free hemorrhage [6]. Severe pulmonary hypertension deserves particular emphasis, as chronic pressure overload induces medial hypertrophy and intimal fibrosis that paradoxically reduces vascular wall compliance, increasing susceptibility to mechanical injury. Anticoagulation compounds this further by eliminating the physiologic tamponade of early perivascular hematoma, facilitating pseudoaneurysm expansion. Left untreated, PAPA carries a recurrent hemorrhage rate of approximately 30-40% with an associated mortality of 40-70% in cases complicated by rupture, as reported in a review of 41 Swan-Ganz catheter-related PAPA cases [5]. 

The clinical presentation of PAPA is variable, ranging from incidental discovery on imaging to massive, life-threatening hemoptysis [5,6]. A critical diagnostic pitfall is attributing post-catheterization hemoptysis to anticoagulation-related bleeding rather than to structural vascular injury, potentially delaying diagnosis. In this case, the therapeutic INR of 2.8 at the time of hemoptysis appropriately raised suspicion for a structural cause rather than over-anticoagulation. CTA of the chest is the diagnostic modality of choice, providing high-resolution visualization of the pseudoaneurysm sac, its feeding vessel, and associated parenchymal changes. Repeat CTA should be performed promptly when new hemoptysis develops following PAC installation, as a pseudoaneurysm may be absent on initial imaging and develop as an interval finding, as demonstrated in this case. 

Endovascular embolization has supplanted surgical intervention as the preferred treatment for PAPA based on available evidence from case series and retrospective studies, demonstrating high technical success rates and low procedural morbidity [8,9]. In a single-center retrospective series from Yale University, coil embolization achieved persistent occlusion in all treated patients at a mean follow-up of 5.3 months with no major adverse events [8]. A bicentric series of eight patients with PAPA, the majority iatrogenic and following Swan-Ganz catheterization, reported 100% technical success [9]. In the present case, a sandwich technique was employed: coil packing of the pseudoaneurysm sac followed by proximal and distal embolization of the supplying segmental artery. Alternative embolic agents include vascular plugs and polymeric agents such as n-butyl cyanoacrylate glue, which may provide faster hemostasis in anticoagulated patients [10]. The choice of embolic agent and technique should be individualized based on pseudoaneurysm anatomy, patient stability, available expertise, and associated complications, with multidisciplinary decision-making guiding the approach. Surgery remains a viable option in cases complicated by hemothorax, failed endovascular therapy, or anatomically inaccessible pseudoaneurysms.

## Conclusions

Iatrogenic PAPA is a rare but potentially fatal complication of Swan-Ganz catheterization. New hemoptysis following placing of PAC, particularly in patients with severe pulmonary hypertension, anticoagulation, advanced age, or female sex, should prompt repeat CTA evaluation. Endovascular treatment, most commonly coil embolization, is the preferred initial approach in most cases, offering definitive pseudoaneurysm exclusion with high technical success and low morbidity, with the optimal strategy guided by individualized assessment and multidisciplinary decision-making.

## References

[1] Rodriguez Ziccardi M, Khalid N (2023). Pulmonary artery catheterization. StatPearls [Internet].

[2] Fukano K, Iizuka Y, Nishiyama S, Yoshinaga K, Uchino S, Sasabuchi Y, Sanui M (2023). Characteristics of pulmonary artery catheter use in multicenter ICUs in Japan and the association with mortality: a multicenter cohort study using the Japanese Intensive care PAtient Database. Crit Care.

[3] Rudziński PN, Henzel J, Dzielińska Z (2016). Pulmonary artery rupture as a complication of Swan-Ganz catheter application. Diagnosis and endovascular treatment: a single centre's experience. Postepy Kardiol Interwencyjnej.

[4] Kalra A, Heitner S, Topalian S (2013). Iatrogenic pulmonary artery rupture during Swan-Ganz catheter placement--a novel therapeutic approach. Catheter Cardiovasc Interv.

[5] Nellaiyappan M, Omar HR, Justiz R, Sprenker C, Camporesi EM, Mangar D (2014). Pulmonary artery pseudoaneurysm after Swan-Ganz catheterization: a case presentation and review of literature. Eur Heart J Acute Cardiovasc Care.

[6] Park HS, Chamarthy MR, Lamus D, Saboo SS, Sutphin PD, Kalva SP (2018). Pulmonary artery aneurysms: diagnosis & endovascular therapy. Cardiovasc Diagn Ther.

[7] Suri P, Kitley C (2022). Successful percutaneous embolization of an intraoperative Swan Ganz catheter-related pulmonary artery injury. Radiol Case Rep.

[8] Fish A, Sailer A, Pollak J, Schlachter T (2023). Pulmonary artery pseudoaneurysms: a single-center experience of endovascular occlusion. CVIR Endovasc.

[9] Fontana F, Piacentino F, Curti M (2023). Pulmonary artery pseudoaneurysms embolization: bicentric experience and review of the literature. J Clin Med.

[10] Truong QA, Massaro JM, Rogers IS (2012). Reference values for normal pulmonary artery dimensions by noncontrast cardiac computed tomography: the Framingham Heart Study. Circ Cardiovasc Imaging.

[11] Humbert M, Kovacs G, Hoeper MM (2022). 2022 ESC/ERS guidelines for the diagnosis and treatment of pulmonary hypertension. Eur Heart J.

[12] Ierardi AM, Xhepa G, Musazzi AM, De Chiara M, Beghi C, Carrafiello G (2015). Endovascular treatment of a pulmonary artery pseudoaneurysm caused by Swan-Ganz catheter deployment in an anticoagulated patient. BJR Case Rep.

